# Cisplatin effects on guinea pigs: cochlear histology and genotoxicity

**DOI:** 10.1590/S1808-86942011000600009

**Published:** 2015-10-19

**Authors:** Cacineli Marion de Franceschi, Tania Tochetto, Aron Ferreira da Silveira, Mara Rejane Fantinel, Thaís Doeler Algarve

**Affiliations:** 1 MSc in Human Communication Disorders - UFSM, Speech and Hearing Therapy; 2 PhD; Associate Professor of the Federal University of Santa Maria (UFSM) at the Speech and Hearing Therapy Program and Graduate Program in Human Communication Disorders; 3 PhD; Full Professor at the Department of Morphology of the Federal University of Santa Maria (UFSM) and Graduate Program in Human Communication Disorders; 4 Biomedic; MSc Student at the Graduate Program in Toxicological Biochemistry -UFSM; 5 Pharmacist. MSc Student of the Graduate Program in Toxicological Biochemistry - UFSM. Federal University of Santa Maria

**Keywords:** hair cells, auditory, genotoxicity, guinea pigs, histology

## Abstract

**Abstract:**

To understand how the DNA answers to external agents such as cisplatin may be relevant to the diagnosis and treatment of hearing disorders caused by the administration of such drug.

**Objectives:**

To investigate the cisplatin influence on the cochlea and DNA of guinea pigs.

**Material and Methods:**

Experimental study carried out with 12 guinea pigs (Cavia porcellus). The inclusion criterion was the presence of Preyer's reflex and distortion-product otoacoustic emissions. Guinea pigs were divided into two groups: Control Group (CG) - made up of six guinea pigs, to which we administrated saline solution during six consecutive days, intraperitoneally; and a Study Group (SG) - made up of six guinea pigs, to which we administrated cisplatin during six consecutive doses of 3mg/kg/day intraperitoneally. Twenty-four hours after the last administration of cisplatin the guinea pigs were slaughtered, blood samples were collected and the cochleae were removed.

**Results:**

The administration of cisplatin did not cause identifiable changes to the DNA. Histological analysis showed changes in the organ of Corti and spiral ganglion.

**Conclusion:**

Cisplatin causes changes in cochlear histology, such as the loss of the normal microcytoarchitecture of the organ of Corti, and reduction of neurons of the spiral ganglion with cell alterations, however, DNA damage was not detected.

## INTRODUCTION

Cisplatin is a broadly used chemotherapy agent in adults and children, to treat solid tumors, with excellent results; however, it has many side effects^1^,^2^. Cisplatin toxicity is found in the kidneys, central and/ or peripheral nervous systems, bowels, bone marrow and also the organ or Corti^3^.

As it reaches the organ of Corti, cisplatin starts its deleterious actions with the support cells, followed by the outer hair cells, especially in the middle and basal turns, later the stria vascularis and auditory nerve^4^.

During the administration of cisplatin, there is a block of the outer hair cells ionic channels, preventing stimulus transduction and causing hearing loss^5^.

Cisplatin causes changes to the antioxidant system of the cochlea's outer hair cells^3^. Moreover, reactive oxygen species (ROS) are produced, which causes cell death^6^. Oxidative stress happens when there is an increased production of free radicals or when antioxidant mechanisms are impaired^7^.

The Cometa Assay has a broad array of applications, including human biomonitoring, genotoxicology and ecological monitoring, as a tool to investigate damage and repair to the deoxyribonucleic acid (DNA) in response to a number of noxious agents^8^. It still provides valuable information about the intrinsic characteristics of the DNA from individual cells^9^.

To understand DNA responses to external agents, such as cisplatin, may be relevant for prevention, diagnosis and treatment of auditory changes caused by the administration of such drugs.

Thus, the present paper aims at checking the impact cisplatin has on cochlear histology and the DNA of guinea pigs.

## MATERIALS AND METHODS

### Study Outline

Experimental study, in which we studied 12 lab animals *(Cavia porcellus)*, because of ease of handling and cochlear dissection, besides being of simple maintenance and resistant to infections^10^,^11^.

We followed the guidelines from the Guide for Care and Use of Animals in the Laboratory, by the Brazilian College of Experimentation with Animals (COBEA)^12^, and also ethical principles of biosafety.

The inclusion criteria for the guinea pigs in the sample was the presence of the Preyer's Reflex (ear pinna contraction upon a sound stimulus) and distortion product otoacoustic emissions (DPOAE).

The drugs used in the study were:

• Cisplatin: 1 mg/ml concentration;
• Saline solution at 0.9%;
• Halothane.

The guinea pigs were assigned to two groups:

**Control Group (CG)** – made up by six lab animals, to which a 0.9% saline solution was intraperitoneally injected during 6 consecutive days;
**Study Group (SG)** – made up by six lab animals, to which six consecutive doses of 3mg/kg/day of cisplatin was intraperitoneally injected.

Hearing was assessed by studying the Preyer reflex and DPOAE before onset and 24 hours after the end of the cisplatin injections. In order to test the Preyer reflex, we used an agogô instrument, large bell, with intensity higher than 90 dB. The guinea pigs should have ear pinna contraction. In order to evaluate the DPOAE, the guinea pigs were sedated with halothane. DPOAE were recorded using a SmartEp otoacoustic emissions device, from *Intelligent Hearing Systems®* (IHS). We analyzed otoacoustic emissions in the frequencies of 500 Hz and 6,000 Hz. Results were printed for analysis and comparison purposes.

The cochlear functional integrity criterion was the very presence of DPOAE^13^, which were also assessed by comparing the mean values of the signal/noise ratio by frequency at the beginning and end of the experiment, in order to observe rates of early suffering of the outer hair cells^14^.

The DNA damage analysis was carried out by the Cometa Assay. In order to do that, we collected blood samples after cisplatin administration.

The Cometa Assay was carried out according to the method proposed by Singh et al.^15^ and modified by Collins et al.^16^. All steps were executed without direct light in order to preclude additional damage to the DNA.

We prepared two slides for each guinea pig, in which we counted 50 nuclei per slide. Both slides were analyzed by two independent examiners. Concerning the damage index (DI), we considered the mean value of the damage observed by the two examiners for 100 nuclei per guinea pig.

**Figure 1:**
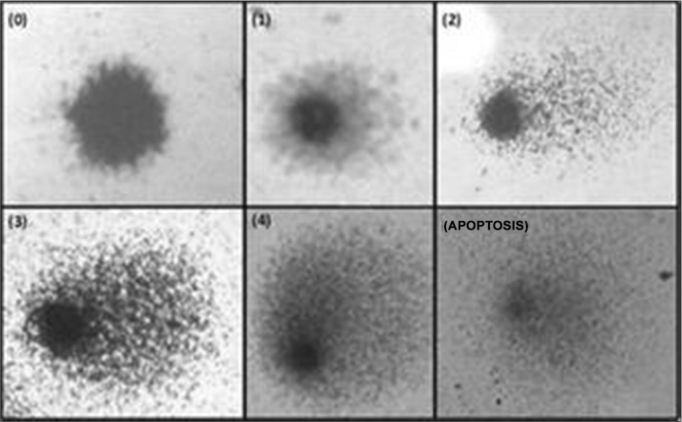
DNA damage classification of the guinea pigs analyzed (Fronza, 2010)^17^.

The “cometa” cells, so called because they have the shape of a comet, dyed with silver nitrate, were analyzed in a regular light microscope and classified according to the size of the tail in relation to the head (Figure 1)^8^:

• Class 0 (ID0): without a tail (no damage);
• Class 1 (ID1): with a small tail, smaller than the head diameter;
• Class 2 (ID2): the tail length is one to two times the diameter of the head;
• Class 3 (ID3): with a tail longer than twice the head diameter;
• Class 4 (ID4): longer and spreader tail (resembling a fan) than class 3;
• Apoptosis: no nucleus.

After the last injection of cisplatin in the SG guinea pigs and saline solution in the CG animals, immediately after collecting the DPOAE, the animals were anesthetized with intramuscular 2% xylazine chlorhydrate and 10% ketamine, and were slaughtered by beheading.

Their temporal bones were quickly removed and open to expose the cochlea, with a median longitudinal cut in the skull, extending posteriorly, towards the vestibule-cochlear region.

In order to avoid cell destruction by autolysis or bacteria and to preserve tissue morphology and composition, the cochleas were fixed with 4% formaldehyde in a buffer solution (pH 7.5). After fixation, we dehydrated the material in growing concentrations of 70% pure ethanol. In order to encase it in paraffin we used xylol so as to make the material translucid. Following that, we used a little fused paraffin and let it cure at room temperature, forming a block with the cochlea inside. Afterwards, the blocks were cut using a steel razor blade coupled to a microtome, from which we obtained 6 to 8μm thickness slices. We used double dyeing by hematoxylin and eosin to color the slides with shades of blue and red^18^.

We used a binocular light Olympus CX40 microscope to inspect the cochlear material and the DNA.

The results from the light microscopy were photographed and analyzed.

The project was approved by the Internal Ethics Committee in Animal Experimentation of the UFSM.

### Statistical analysis

Data normality was tested by means of the Kolmogorov-Smirnov test, which determines whether the characteristic in the sample studied comes from a population with normal distribution.

When the normality of the variables in the study was tested, we noticed that all tested data followed a normal distribution.

We used the T-*Student* test to analyze the ears (right and left), the mean value of the DPOAE amplitudes and the Cometa Assay. *P* values below 0.05 (*p*<0.05) were considered statistically significant.

In order to calculate the damage index (DI), we used the following formula, according to Cavalcanti et al.^19^.

ID= (0 x n0) + (1 x n1) + (2 x n2) + (3 x n3) + (4 x n4), where n = number of nuclei from each class analyzed.

Thus, the index of damage for 100 nucleotides may vary from zero (totally undamaged 0 X 100) to 400 (totally damaged 100 X4).

We used the Spearman correlation coefficient in order to correlate the DNA with the damage index. The Spearman's ρ coefficient varies between -1 and 1. The closer it is to these extremes, the greater will be the association between the variables. The negative correlation sign means that the variables vary in the opposite direction. 0.70 (+ or -) points to a strong correlation. A significant correlation was considered at the level of 0.001.

## RESULTS

The animals were divided in two groups: CG (Control Group) are the ones which received saline solution; and the SG (Study Group) are the ones injected with cisplatin. Each group had six guinea pigs.

We did not find statistically significant differences between right and left ear behavior when they were analyzed using the T-*Student test.* Therefore, the data obtained from both ears were studied together.

We found the Preyer' reflex and the DPOAE in all guinea pigs from the CG and the SG, before we started to inject the saline solution and cisplatin, respectively, making up a total of 24 ears.

After six days injecting cisplatin, four SG animals died. It was possible to remove the eight cochleas for histology analysis and from one of them we managed to collect blood samples for DNA analysis, as well as in the remaining guinea pigs.

There were no statistically significant differences in the Cometa Assay among the guinea pigs from the CG and the SG (Table 1).

**Table 1:** DNA damage in the control and study groups.

Damage Index	Control		Study		*p*-value
	Mean	Standard deviation	Mean	Standard deviation	
DI0	41.37	2.76	39.58	3.41	0.420519
DI1	4.93	1.84	5.08	1.58	0.906553
DI2	1.72	0.72	2.66	0.28	0.073190
DI3	1.19	0.64	1.25	0.90	0.917171
DI4	1.59	1.20	2.33	0.72	0.371587
APOPTOSIS	2.50	2.65	5.16	4.40	0.284619

(p-value<0.005 or p-value lower than 5%)

DI- Damage Index

In CG animals, the injection of 0.9% saline solution resulted in no or very little damage to the DNA, that is, there was a highly negative correlation (-0.943*) among the DI0 variables (no damage, no tail) and DI1 (with little damage, having a tail smaller than the head's diameter) (*significant correlation at 0.001).

In the SG animals, the injection of 3mg/kg of cisplatin for six consecutive days caused little or total DNA damage, in other words, three was a highly negative correlation (-1.00*) among the apoptosis variables (totally damaged, without the nucleus) and DI1 (with little damage, with a tail smaller than the head's diameter) (*significant correlation at the level of 0.001).

**Figure 2:**
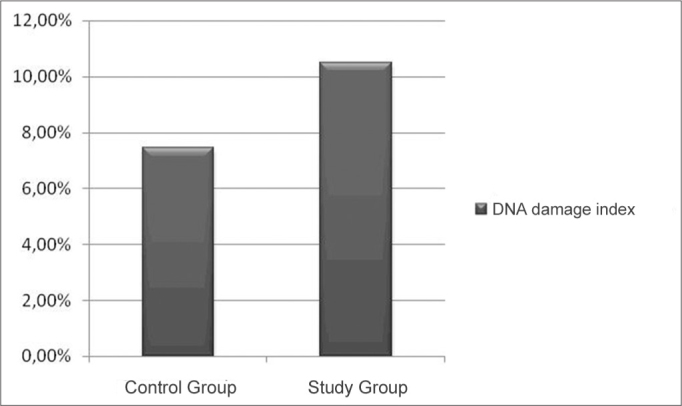
DNA damage index per group.

The DNA damage index in the CG (7.45%) was lower than that of the guinea pigs from the SG (10.5%) (Figure 2).

**Figure 3:**
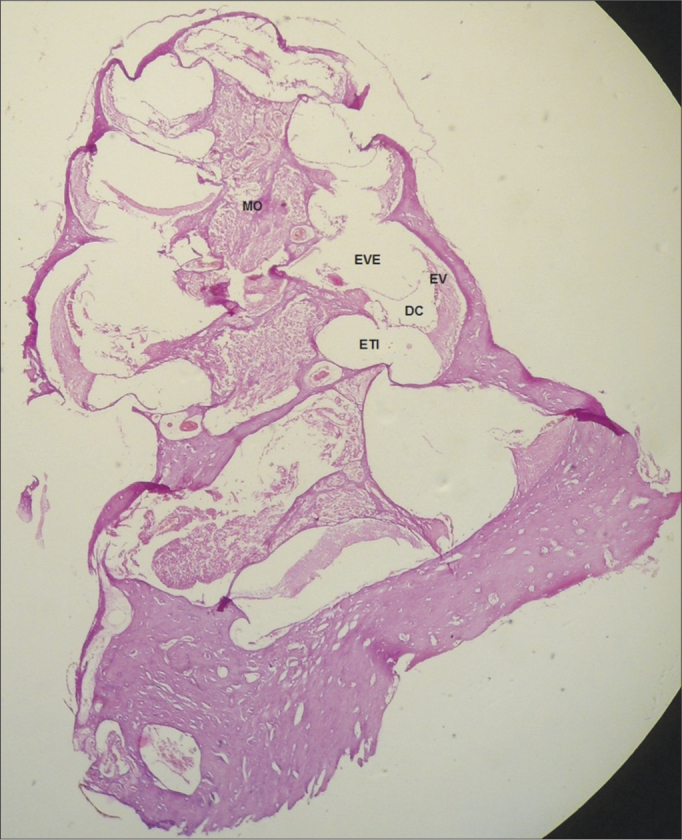
Microscopic structure of the guinea pig cochlea from the CG in the cross-section from base to apex, the modiolus in the center, within which we find spiral ganglion neurons. 4x magnification. MO - Modiolus; EVE – Scala Vestibuli; ETI – Scala Tympani; DC – Cochlear Duct; EV – Stria Vascularis.

**Figure 4:**
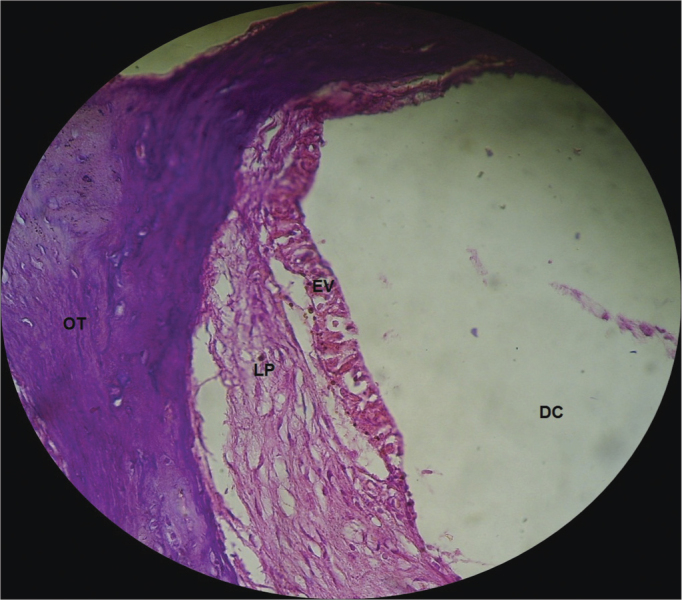
Normal looking stria vascularis, without changes in a guinea pig from the CG. 40x magnification. DC – Cochlear Duct; EV – Stria Vascularis; LP - Lamina Propria; OT – Temporal Bone.

**Figure 5:**
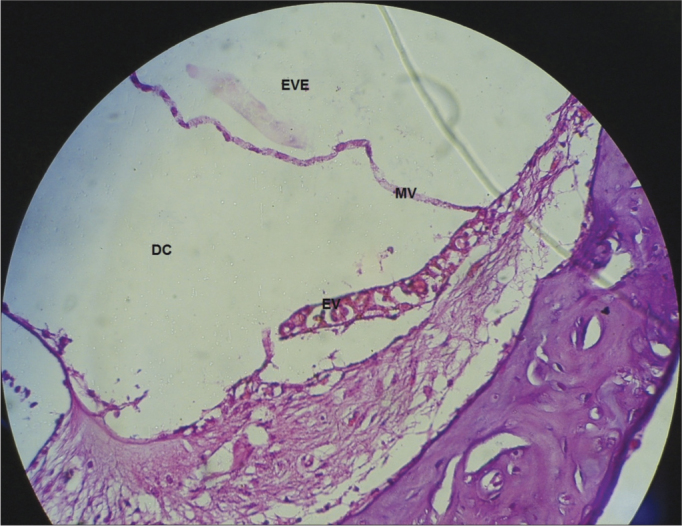
Stria vascularis without vacuolization in a guinea pig from the CG. 40x magnification. MV – Vestibular Membrane; EVE – Scala Vestibuli; DC – Cochlear Duct; EV – Stria Vascularis.

**Figure 6:**
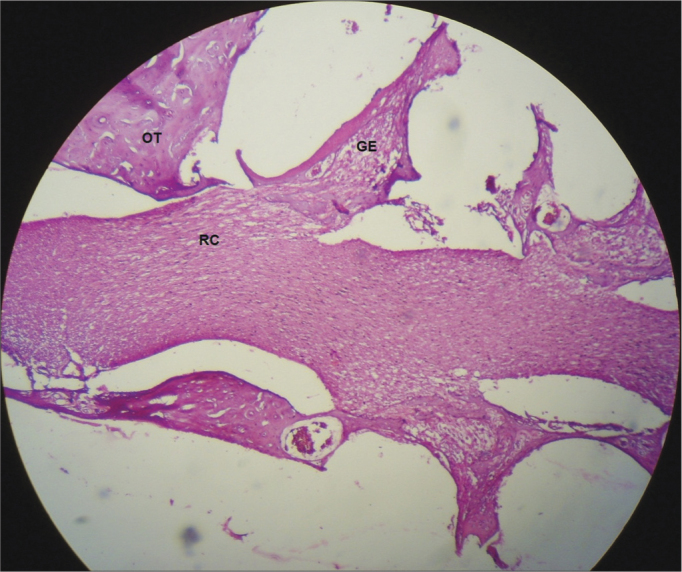
Start of the cochlear branch, showing normal aspects and without demyelination in a CG guinea pig. 10x magnification. OT – Temporal Bone; GE – Spiral Ganglion; RC – Cochlear Branch.

**Figure 7:**
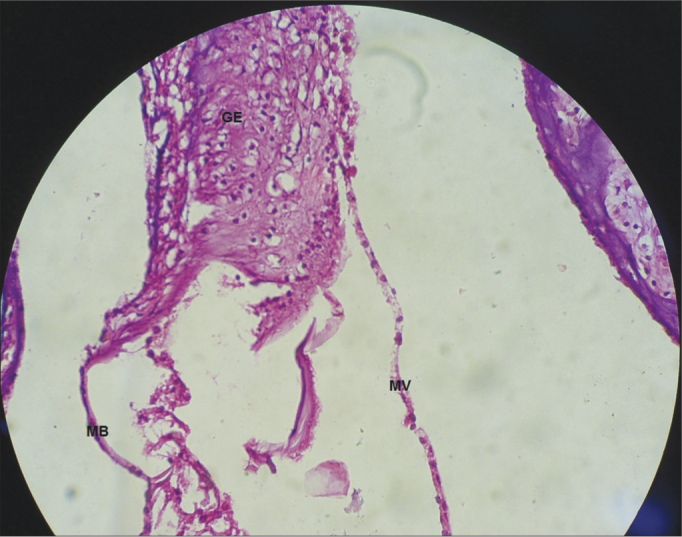
Vestibular membrane without vacuolization, intact and straight from a CG guinea pig. 40x magnification. MV – Vestibular Membrane; MB – Basilar Membrane; GE – Spiral Ganglion.

**Figure 8:**
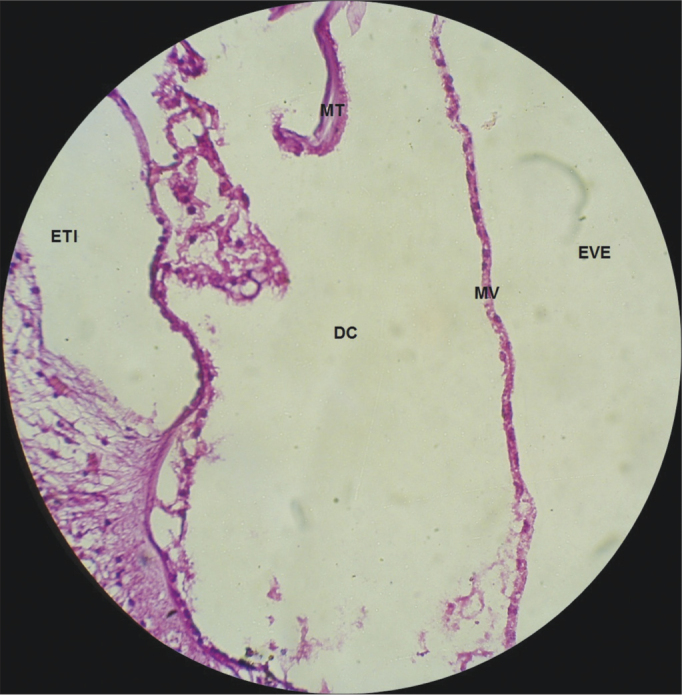
Vestibular membrane without vacuolization, intact and straight, in a CG guinea pig. 10x magnification. MV – Vestibular Membrane; MT – Tectorial Membrane; EVE – Scala Vestibuli; ETI – Scala Tympani; DC – Cochlear Duct.

In the CG guinea pigs which received the 0.9% sodium chloride solution, the cochlear microscopic structure in a cross section from the basis to the apex, it showed the turns, the modiolus in the center, within which we found the spiral ganglion neurons (Figure 3); the stria vascularis without changes (Figures 4 and 5); the cochlear branch looked normal, without demyelination (Figure 6); the spiral ganglion neurons did not show any changes, a straight line and without cytoplasmic vacuolization (Figures 7 and 8).

**Figure 9:**
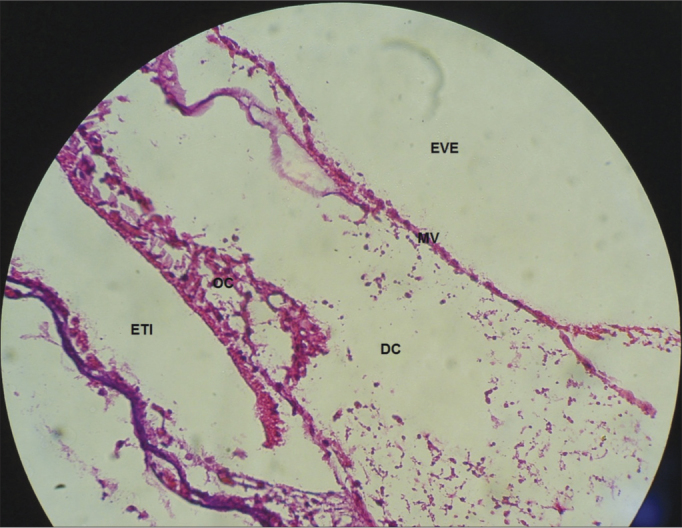
Extensive loss of the normal microarchitecture of the organ of Corti, outer and inner hair cells without definition. No tectorial membrane in a guinea pig treated with cisplatin at the dose of 3mg/kg for six consecutive days. 40x magnification. MV – Vestibular Membrane; EVE – Scala Vestibuli; ETI – Scala Tympani; DC – Cochlear Duct; OC – Organ of Corti.

**Figure 10:**
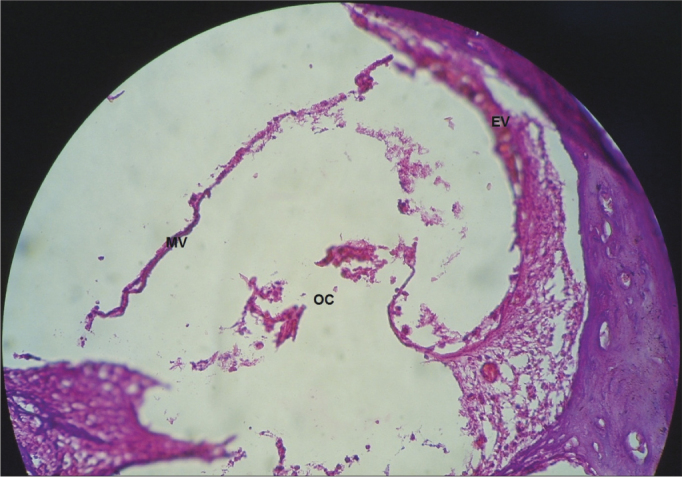
Total absence of the Organ of Corti in the middle cochlear turn of the guinea pigs injected with cisplatin at the dose of 3mg/kg for six consecutive days. 40x magnification. MV – Vestibular Membrane; EV – Stria Vascularis; OC – Organ of Corti.

**Figure 11:**
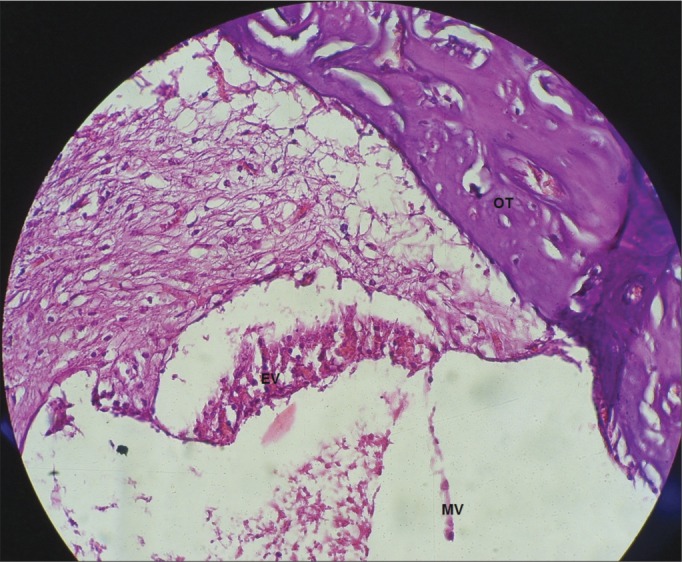
Generalized changes in the stria vascularis cells, without definition of cell organization and vestibular membrane aspect from the guinea pigs treated with 3mg/kg of cisplatin for six consecutive days. 40x magnification. MV – Vestibular Membrane; EV – Stria Vascularis; OC – Organ of Corti.

**Figure 12:**
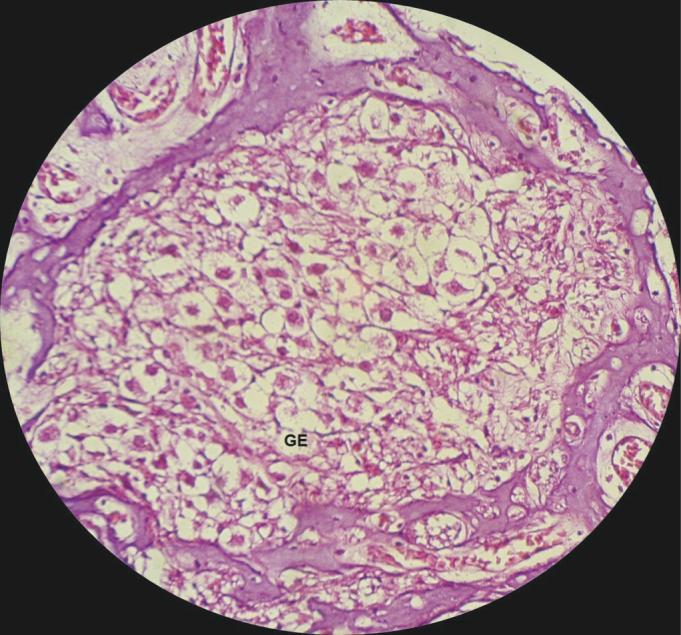
View of the spiral ganglion neurons from a guinea pig injected with 3mg/kg of cisplatin for six consecutive days. 40x magnification. GE – Spiral Ganglion.

In the guinea pigs which received cisplatin (SG), we found an extensive loss of the normal microarchitecture of the organ of Corti (Figure 9): outer and inner hair cells without definition (Figure 9); no tectorial membrane (Figure 9); complete absence of the organ of Corti in the middle turn of the cochlea (Figure 10); generalized change in the stria vascular cells, without cell definition (Figure 11); broken vestibular membrane and many cells do not have the nucleus (Figure 11); scattered spiral ganglion neurons with cell changes, such as lack of nucleus (Figure 12).

## DISCUSSION

There were no statistically significant differences between the Cometa Assay test groups (Table 1). In fact, cisplatin does not produce damage detectable by the Cometa Assay because of cross connections which impact DNA migration^20^, ^21^, ^22^, ^23^, ^24^.

In order to analyze the DNA from the CG guinea pigs, there was a highly negative correlation among the DI0 and DI1 variables. The higher the DI0 the lower the DI1, and vice-versa, in other words, there are no high rates of DNA damage to the CG animals.

In SG guinea pigs there was a highly negative correlation between the apoptosis and DI1 variables, in other words, as the number of apoptosis reduces, the DI1 drops, and vice-versa, thus, it is possible to see that, in the SG guinea pigs, there was low DNA damage index, or the cell is destroyed. Slattery & Warchol^25^ reported that the treatment with cisplatin causes apoptosis of auditory and vestibular hair cells.

DNA damage index was low both in the CG as well as in the SG. In the CG animals, the DNA damage index found was 7.45% (Fig. 2). In the SG animals, the damage index was 10.5% (Fig. 2). There was a mild increase in the DNA damage index in the guinea pigs which received cisplatin.

In the CG animals which received 0.9% saline solution, we noticed that the cochlear microscopic structure in a cross-section from the base to the apex shows the turns, the modiolus in the center, within which we find the spiral ganglion neurons (Figure 3), a normal-looking stria vascularis (Figures 4 and 5), normal looking cochlear branch, without demyelination (Figures 6) and spiral ganglion neurons without changes (Figures 7), normal looking vestibular membrane – straight and without cytoplasmic vacuolization (Figures 7 and 8). Other studies were also unable to find histological changes in the groups which were not treated with cisplatin^26^,^27^.

The low quality of the histology slides made our analysis very difficult. The blocks were made with paraffin, so that the slide quality could be high; the ideal material would have been resin blocks, however, it was not available.

In the guinea pigs which received cisplatin, there were generalized changes in the stria vascularis cells, without cell definition (Figure 11). The stria vascularis is one of the main targets of cisplatin in the cochlea^27^, ^28^, ^29^. Nonetheless, Cardinaal et al.^26^ did not see any change in the stria vascularis of the animals which received cisplatin for eight consecutive days, with the lower daily dose (0.7 mg/kg/day) as the highest daily dose (2.0mg/ kg/day). Other experiments also did not find any histological effect in the stria vascularis (dose of 2mg/kg/day for 4, 6 and 8 consecutive days)^27^, ^28^, ^29^. Thus, it is possible to infer that the dose capable of causing damage to the stria vascularis is higher than 2.0 mg/kg/day.

In the SG guinea pigs, the spiral ganglion neurons were scattered and with cell changes, such as no nuclei (Figure 12). Such findings are in agreement with those from Cardinaal et al.^26^, who found morphological changes in the spiral ganglion cell, such as cytoplasmic vacuolization. Other studies also reported spiral ganglion changes^27^, ^28^, ^29^. We noticed that the spiral ganglion is one of the main targets of the changes caused by cisplatin^27^, ^28^, ^29^.

In the guinea pigs which received cisplatin (SG), 3mg/kg dose for six consecutive days, we noticed an extensive loss of the normal microarchitecture of the organ of Corti (Figure 9), outer and inner hair cells without definition (Figure 9), no tectorial membrane (Figure 9) and complete absence of the organ of Corti in the middle cochlear turn (Figure 10). These findings corroborate those from Cardinaal et al.^26^, who showed a severe loss of outer hair cells, especially in the basal and middle turns of the cochlea. Other studies have also shown changes to the organ of Corti, with extensive damage to the outer hair cells^27^, ^28^, ^29^. Nonetheless, they reported that the basal turn was the most affected one^27^.

Although cisplatin did not cause statistically significant genotoxic changes found in the Cometa Assay, it was possible to see changes in cochlear histology. Such findings corroborate other studies which reported the production of cross-connections which impact DNA migration in the Cometa Assay^20^, ^21^, ^22^, ^23^, ^24^. Thus, we suppose that cisplatin causes changes to the cell antioxidant mechanism. Cisplatin ototoxicity mechanism is due to changes to the cochlear outer hair cell antioxidant mechanisms^3^. Moreover, there is production of oxygen reactive species (ORS), which would cause cell death^6^. Van Ruijven et al.^29^ showed the presence of platinumloaded DNA in the nucleus of most of the organ of Corti cells after the administration of cisplatin. Thus, cisplatin forms cross-connections with the DNA, which prevents DNA migration in the Cometa Assay. However, cisplatin reduces cell antioxidant capacity, causing cell death, which causes the cochlear changes seen upon histological analysis.

## CONCLUSION

Cisplatin causes changes to the cochlear histology such as the loss of the normal micro-architecture of the organ of Corti and reduction of neurons in the spiral ganglion with cell changes. Nonetheless, we did not detect genotoxic damage.
